# Production of Secondary Metabolites in Extreme Environments: Food- and Airborne *Wallemia* spp. Produce Toxic Metabolites at Hypersaline Conditions

**DOI:** 10.1371/journal.pone.0169116

**Published:** 2016-12-30

**Authors:** Sašo Jančič, Jens C. Frisvad, Dragi Kocev, Cene Gostinčar, Sašo Džeroski, Nina Gunde-Cimerman

**Affiliations:** 1 Department of Biology, Biotechnical Faculty, University of Ljubljana, Jamnikarjeva 101, Ljubljana, Slovenia; 2 Department of System Biology, Technical University of Denmark, Søltofts Plads, Building 221, Kgs. Lyngby, Denmark; 3 Department of Knowledge Technologies, Jožef Stefan Institute, Jamova cesta 39, Ljubljana, Slovenia; 4 Centre of Excellence for Integrated Approaches in Chemistry and Biology of Proteins (CIPKeBiP), Jamova 39, Ljubljana, Slovenia; Universita degli Studi di Pisa, ITALY

## Abstract

The food- and airborne fungal genus *Wallemia* comprises seven xerophilic and halophilic species: *W*. *sebi*, *W*. *mellicola*, *W*. *canadensis*, *W*. *tropicalis*, *W*. *muriae*, *W*. *hederae* and *W*. *ichthyophaga*. All listed species are adapted to low water activity and can contaminate food preserved with high amounts of salt or sugar. In relation to food safety, the effect of high salt and sugar concentrations on the production of secondary metabolites by this toxigenic fungus was investigated. The secondary metabolite profiles of 30 strains of the listed species were examined using general growth media, known to support the production of secondary metabolites, supplemented with different concentrations of NaCl, glucose and MgCl_2_. In more than two hundred extracts approximately one hundred different compounds were detected using high-performance liquid chromatography-diode array detection (HPLC-DAD). Although the genome data analysis of *W*. *mellicola* (previously *W*. *sebi sensu lato*) and *W*. *ichthyophaga* revealed a low number of secondary metabolites clusters, a substantial number of secondary metabolites were detected at different conditions. Machine learning analysis of the obtained dataset showed that NaCl has higher influence on the production of secondary metabolites than other tested solutes. Mass spectrometric analysis of selected extracts revealed that NaCl in the medium affects the production of some compounds with substantial biological activities (wallimidione, walleminol, walleminone, UCA 1064-A and UCA 1064-B). In particular an increase in NaCl concentration from 5% to 15% in the growth media increased the production of the toxic metabolites wallimidione, walleminol and walleminone.

## Introduction

Filamentous fungi inhabit a large variety of different ecological habitats [1]. Competition-selected fungi are characterized by their ability to produce secondary metabolites [2, 3] and various extracellular enzymes that can be of interest to the agrochemical, food, and pharmaceutical industries [4, 5]. Fungal secondary metabolites originate from a few common biosynthetic pathways [6] and many of these are not directly involved in initial mycelial growth phase, but may play a crucial role in nutrition, sporulation processes, interactions with other organisms and stress tolerance [7].

The detailed search for the production of secondary metabolites has been focused mainly on cosmopolitan soil borne fungi such as genera *Penicillium* and *Aspergillus* [8–10]. With a few exceptions such as reports on the extrolite production by halotolerant and halophilic fungi from the genera *Aspergillus* and *Penicillium* [11, 12] extremophilic fungi remained mainly overlooked, due to the general opinion that extremophiles need a smaller number of bioactive molecules to interact with a limited number of competing species in extreme environments [11, 13, 14]. Indeed the diversity of secondary metabolites in the investigated stress-tolerant or stress-selected fungi was rather low [15]. For example, no secondary metabolites were identified for the fungus *Xeromyces bisporus* that is able to tolerate the lowest water activity (a_w_ ≈ 0.61) of all microorganisms [16].

On the other hand, rather than just investigate soil microorganisms it has been proposed by several authors to screen unusual or extreme habitats for producers of novel interesting bioactive secondary metabolites. In their opinion fungi in extreme environments might have evolved unique metabolic mechanisms, as response to unusual conditions, with potential implications in drug discovery [11, 17].

The genus *Wallemia* is taxonomically placed in the phylum Basidiomycota, which harbour few extremophilic representatives [18, 19]. This genus was previously described as halophilic; however, it is more appropriate to regard it as xerophilic, since only *W*. *ichthyophaga* grows better at high concentrations of salt than at high concentration of non-ionic solutes [19–21]. As reported for other fungi adapted to low a_w_, *Wallemia* spp. can contaminate food preserved with either high amounts of salt or sugar, or desiccation [20, 22, 23]. Some foods, such as dry cured meat products, contain high concentrations of NaCl due to the production process and are typical habitats of *Wallemia* spp., particularly *W*. *ichthyophaga* [19, 23].

Currently, *Wallemia* comprises seven species, of which *W*. *sebi*, *W*. *mellicola* and *W*. *muriae* are very common and can often be isolated from indoor and outdoor air, whereas the remaining species (*W*. *ichthyophaga*, *W*. *hederae*, *W*. *canadensis*, and *W*. *tropicalis*) are rare and occupy specific habitats, such as brine in salterns [19, 23–25].

Until 2005 *W*. *sebi* represented the only known species of the genus *Wallemia* [19], thus reports regarding secondary metabolites (**Table 1**) were limited only to this species. *Wallemia sebi* was reported to produce several bioactive metabolites, such as toxic wallimidione [26], walleminone and walleminol [27–29], UCA 1064-A and UCA 1064-B [30, 31] and pigments [32, 33]. Walleminol (named also walleminol A) has been detected in food (jam and cake), both naturally and artificially contaminated with *W*. *sebi* [29]. Walleminol has an LD_50_ of 40 μg mL^-1^ for brine shrimp and *Tetrahymena pyriformis*, and a minimum inhibitory dose of 50 μg mL^-1^ for rat liver cells and baby hamster kidney cells [27]. Walleminol is usually difficult to detect [26], due to natural interconversion to walleminone [28], previously designated as walleminol B [27]. In Toxtree the toxicity of walleminone was predicted ad modest [26]. UCA 1064-A and UCA 1064-B exhibit antitumor activity against mouse mammary tumour model, antifungal activity against *Saccharomyces cerevisiae*, antimicrobial activities against Gram-positive bacteria at 40 μg mL^-1^, and antiproliferative activities against HeLa S3 cells. Their toxic effects are not known [31].

**Table 1:** Secondary metabolites reported from the *Wallemia*.

Component	Elementary composition	Monoisotopic mass (Da)
3-methylfuran (Peng et al. 2011)	C_5_H_6_O	82.04
4-methylfuran-2-carboxylic acid (Peng et al. 2011)	C_6_H_6_O_3_	126.03
5-methyluracil = thymine (Peng et al. 2011)	C_5_H_6_N_2_O_2_	126.04
2,5-furandimethanol (Peng et al. 2011)	C_6_H_8_O_3_	128.05
5-methylpyridin-3-ol (Peng et al. 2011)	C_6_H_8_O_3_	128.05
phenylacetic acid (Desroches et al. 2014)	C_8_H_8_O_2_	136.05
*p*-hydroxybenzoic acid (Desroches et al. 2014)	C_7_H_6_O_3_	138.03
5-hydroxy-3-coumaranone (Peng et al. 2011)	C_8_H_6_O_3_	150.03
**Tryptophol (Wood et al. 1990)(Desroches et al. 2014)**	**C**_**10**_**H**_**11**_**NO**	**161.08**
3-hydroxy-5-methyl-5,6-dihydro-7-cyclopentapyridin-7-on^a^ (Peng et al. 2011)	C_9_H_9_NO_2_	163.06
3-(hydroxyacetyl)indole (Peng et al. 2011)	C_10_H_9_NO_2_	175.06
Indole acetic acid (Peng et al. 2011)	C_10_H_9_NO_2_	175.18
(S)-3-hydroxy-4-(4-hydroxyphenyl)-2-one (Peng et al. 2011)	C_10_H_13_O_3_	181.09
(2s, 3s)-1-(4-hydroxyphenyl) butan-2,3-diol (Peng et al. 2011)	C_10_H_14_O_3_	183.09
(2R, 3S)-1-(4-hydroxyphenyl) butan-2,3-diol (Peng et al. 2011)	C_10_H_14_O_3_	183.09
Tryptophol acetate (Desroches et al. 2014)	C_12_H_13_NO_2_	203.09
Walleminol = Walleminol A^a^ (Wood et al. 1990)(Frank et al. 1999)	C_15_H_24_O_2_	236.18
Wallimidione^a^ (Desroches et al. 2014)	C_14_H_16_N_2_O_2_	244.12
Walleminone = Walleminol B (Wood et al. 1990)(Frank et al. 1999)(Desroches et al. 2014)	C_15_H_24_O_3_	252.17
Wallemia C (Badar et al. 1973)(Ito et al. 1981)	C_17_H_19_NO_2_	269.14
Wallemia A (Badar et al. 1973)(Ito et al. 1981)	C_17_H_21_NO_2_	271.16
Wallemia F (Badar et al. 1973)(Ito et al. 1981)	C_17_H_18_ClNO_2_	303.1
**Wallemia E (Badar et al. 1973)(Ito et al. 1981)**	C_17_H_20_ClNO_2_	305.12
UCA 1064-A = A25822B^a^ (Chamberlin et al. 1974)(Takahashi et al. 1993)	C_28_H_45_NO	411.35
UCA 1064-B^a^ (Takahashi et al. 1993)	C_28_H_47_NO	413.37

^a^ metabolites with significant biological activities (e.g., toxic, antibacterial, antifungal, antitumor, antiproliferative)

All above described compounds were discovered in the *W*. *sebi* culture grown on media suitable for “mesophilic” fungi (a_w_ ≈ 1.0), while cyclopentanopyridine alkaloid, together with 11 aromatic compounds was isolated during growth of *W*. *sebi* in medium with 10% NaCl (w/v) [17]. This compound exhibited antibacterial activity against *Enterobacter aerogenes* [17]. The acetone extracts of *W*. *sebi* and *W*. *ichthyophaga* grown at 10% NaCl (w/v) exhibited antibacterial activity against a Gram positive bacterium *Bacillus subtilis* [34]. A complex mixture of 21 sterols and fatty acids was discovered in the ethanol extract of *W*. *sebi* grown at 5% and 20% NaCl (w/v), which were presumed to be responsible for the haemolytic activity of the extract [35].

In terms of food safety, the aim of the current work was to perform a comparative investigation of the secondary metabolites produced by the seven species of the food- and airborne genus *Wallemia* in osmotically unstressed and stressed conditions. Production of secondary metabolites was investigated by high-performance liquid chromatography-diode array detector (HPLC-DAD). To define key features influencing the production of secondary metabolites in *Wallemia*, the obtained dataset was analysed with machine learning. Another aim was to use mass spectrometry to investigate the production of biologically significant secondary metabolites such as wallimidione, walleminol, walleminone, UCA 1064-A and UCA 1064-B in all *Wallemia* species across the salinity gradient.

## Materials and Methods

### AntiSMASH and other genomic analyses

Secondary metabolite biosynthesis clusters in the genome sequences of *W*. *mellicola*, previously identified as *W*. *sebi* [36], and *W*. *ichthyophaga* [37] were identified by the antiSMASH 2.0 (Antibiotics and Secondary Metabolites Analysis Shell; http://antismash.secondarymetabolites.org/) pipeline [38]. The antiSMASH can identify biosynthetic clusters that cover the whole range of known secondary metabolite compound classes [39]. Nucleotide sequences of the genomes of *W*. *ichthyophaga* and *W*. *mellicola* were uploaded to the antiSMASH server in FASTA format. Analysis was performed for DNA of eukaryotic origin; other settings were left at their default values.

Predicted proteomes were searched for the homologues of known polyketide synthases (PKSs) and non-ribosomal peptide synthetases (NRPSs) involved in the biosynthesis of mycotoxins [40] with standalone psiblast (included in blast 2.2.25+; Altschul et al. [41]) with the e-value cut-off at 10^−10^. The recovered hits from *W*. *ichthyophaga* and *W*. *mellicola* were blasted back to SwissProt and non-redundant NCBI (NCBI-NR) databases using the same parameters as above. The most similar 100 hits of each protein were recovered and a phylogenetic analysis was performed with the PhyML 3.1 software [42]. Approximate Bayes (aBayes) branch supports were calculated. The analysis was run using the model of evolution, α-parameter of the γ-distribution of six substitution rate categories, and the determined proportion of invariable sites as estimated by the ProtTest [43]. The function of the proteins from *W*. *ichthyophaga* and *W*. *mellicola* was predicted according to their phylogenetic position in regard to proteins with known functions recovered from SwissProt and/or NCBI-NR.

### Cultures, media and growth conditions

The analysis included 30 strains of seven *Wallemia* spp. (5 of *W*. *ichthyophaga*, 3 of *W*. *hederae*, 5 of *W*. *muriae*, 5 of *W*. *sebi*, 5 of *W*. *mellicola*, 3 of *W*. *tropica*lis and 4 of *W*. *canadensis*). They originated from low a_w_ foods (maple syrup, chocolate, jam, salted ham, barley), indoor (air, dust, wall), soil (peat soil), plants (seeds, pollen) and hypersaline environments (hypersaline water of salterns, sea salt, bitterns) (**S1 Table**). Strains were obtained from the CBS-KNAW Fungal Biodiversity Centre, Utrecht, The Netherlands [CBS]; Canadian Collection of Fungal Cultures, Agriculture and Agri-Food Canada, Ottawa, Canada [CCFC/DAOM]; the Ex Culture Collection of the Department of Biology, Biotechnical Faculty, University of Ljubljana, Infrastructural Centre Mycosmo, MRIC UL, Ljubljana, Slovenia [EXF]; the Mycotheque of the Catholic University of Louvain, Louvain la Neuve, Belgium [MUCL]; and the University of Alberta Microfungus Collection and Herbarium, Edmonton, Canada [UAMH]). The strains were identified as part of the previous taxonomic revisions of the genus *Wallemia* spp. [19, 23, 24].

Spore suspensions were prepared in sterile spore suspension medium [44] from the cultures grown on MY50G [20] for *W*. *hederae*, *W*. *muriae*, *W*. *sebi*, *W*. *mellicola W*. *tropica*lis, *W*. *canadensis* or MEA + 17% NaCl (w/v) [45] for *W*. *ichthyophaga* at 25°C for 10 days in the dark. For the secondary metabolite production the following media were used for nine point inoculation: (i) YES (Difco yeast extract sucrose agar; a_w_ ≈ 1.00) [22], (ii) CYAS (Czapek yeast autolysate agar with 50 g/L NaCl; a_w_ ≈ 1.00) [44] and (iii) WATM (Wickerhams antibiotic test medium) [46]. WATM was supplemented with sucrose (20% [a_w_ ≈ 0.98], 55% [a_w_ ≈ 0.90], 65% [a_w_ ≈ 0.86], 70% [a_w_ ≈ 0.80]; all w/v), NaCl (5% [a_w_ ≈ 0.98], 15% [a_w_ ≈ 0.90], 20% [a_w_ ≈ 0.86], 25% [a_w_ ≈ 0.80]; all w/v) and MgCl_2_ (4% [a_w_ ≈ 0.98], 11% [a_w_ ≈ 0.90], 17% [a_w_ ≈ 0.86]; all w/v). WATM contains corn steep liquid as an additional nitrogen source, known to support the satisfactory production of secondary metabolites [44]. All media were autoclaved for 15 min at 121°C. Strains were grown at 25°C for 10 days in the dark.

According to growth of individual species across the NaCl, glucose and MgCl_2_ concentration ranges [23, 24, 47, 48], several concentrations of each solute for each of the species were selected for screening (**S1 Table**).

### Extraction and HPLC-DAD analysis

Culture extracts were prepared by excising six colony plugs of 6 mm diameter from six colonies using a cork drill. Each plug consisted from fungal colony and agar medium bellow the colony. Plugs from un-inoculated plates were used as a negative control and processed in the same way as other samples. The plugs were pooled into a 1.5 mL screw cap vial. A mixture of methanol-dichloromethane-ethyl acetate (1:2:3) containing 1% formic acid was added (500 μL) prior to ultrasonic extraction for 60 min [49]. The organic phase was transferred to clean vials and dried by centrifugation under vacuum. The residue was re-dissolved in 500 μL methanol and filtered through 0.45 μm filter (Sartorius). One μl of the solution was subjected to HPLC analysis (Chromeleon Dionex UHPLC with a Dionex Ultimate 3000 RS Diode array detector) adopting alkylphenone retention indices and diode array UV-VIS detection from 200–600 nm or, respectively, at 210 nm for the detection of separated secondary metabolites. Separations were done on a 2 × 100 nm Luna2 OOD-4251-BO-C_18_ column with a C_18_ pre-column, both packed with 3 μm particles. A linear gradient starting from 85% water and 15% acetonitrile going to 100% acetonitrile in 20 min, then maintaining 100% acetonitrile for 5 min, was used at a flow rate of 0.4 mL min^–1^. Both eluents contained 0.005% trifluoroacetic acid (TFA). The compounds were identified by their retention times and UV/VIS spectra. All peaks were quantified by height and collected in an Excel file for machine learning. We attempted to identify as many secondary metabolites as possible, the rest were given provisional names. Peaks detected in medium extracts were excluded from the analyses of culture extracts.

In addition, elementary composition and monoisotopic mass of selected compounds was performed on an Agilent Infinity 1290 UHPLC System (agilent Technolgies, Santa Clara, CA, USA), equipped with a diode array detection, as detailed in Kildgaard, Mansson (50).

### Machine learning methods

The HPLC-DAD analyses produced a dataset that was used for performing data mining. Each of the samples was described with the species itself (*W*. *hederae*, *W*. *ichthyophaga*, *W*. *muriae*, *W*. *sebi*, *W*. *mellicola*, *W*. *canadensis*, *W*. *tropicalis*), solute used for lowering the water activity of the medium (NaCl, glucose, MgCl_2_ or none), the concentration of the solute (for NaCl: 5%, 15% and 20%; for glucose: 20%, 55%, 65% and 70%; for MgCl_2_: 4%, 11% and 17%; all w/v) and identified secondary metabolites. The species, the type of solute and the concentration of the solute were used as descriptive variables, while the quantities of the secondary metabolites were used as target variables.

The generic data analysis task that we addressed was a task of predictive modelling, relating the descriptive variables with the target variables. In other words, we were trying to find which variables contributed to the differences in the production of secondary metabolites. We defined three different scenarios for the analysis based on the use of different descriptive variables. The analysis scenarios were as follows: A) the species itself is the only descriptive variable, B) both the species itself and the a_w_ (concentration of the solute) are the descriptive variables, and C) the type and the concentration of the solute are the descriptive variables. Furthermore, we repeated the scenario C for each of the species separately, thus obtaining 7 additional models.

To analyse the data, we used the machine learning tool CLUS available for download at http://clus.sourceforge.net. More specifically, predictive clustering trees (PCTs) for multi-target regression were used as models. PCTs are a generalization of regression trees–a machine learning approach commonly used for regression. PCTs, similarly as regression trees, are a tree-like structure that have internal nodes and leafs. The internal nodes contain tests on the descriptive variables, while leafs represent the predictions of the target variables. PCTs can solve the more general task of structured output prediction, including the task of multi-targeted regression [50–53].

### Mass spectrometry

To investigate the most biologically significant metabolites (wallimidione, walleminol, walleminone, UCA 1064-A and UCA 1064-B), 30 extracts that showed the most diverse profile of secondary metabolites according to the HPLC-DAD analysis were selected. All analysed extracts were obtained from the fungal mycelium grown on media with 0%, 5%, 15% and 20% NaCl (w/v). Mass spectroscopy was performed using a protocol described by Nielsen et al. [10] and Kildgaard et al. [54].

## Results and Discussion

### Low number of secondary metabolites clusters in the genomes of *W*. *mellicola* and *W*. *ichthyophaga*

Genomic analyses have indicated that genes involved in the fungal synthesis of secondary metabolites are frequently found in contiguous clusters [6]. For example, 66, 38, 70 and 73 secondary metabolite biosynthetic gene clusters were predicted in *Aspergillus nidulans*, *A*. *fumigatus*, *A*. *niger* and *A*. *oryzae*, respectively [55]. Genomic analyses also revealed that the ability of fungi to produce secondary metabolites has been substantially underestimated, because many of the fungal secondary metabolite biosynthesis gene clusters are not expressed under standard cultivation conditions [56].

Although a substantial number of secondary metabolites were detected in the cultures of *Wallemia* spp., only nine secondary metabolite clusters were identified in the genome of *W*. *mellicola* (previously identified as *W*. *sebi*; Jančič et al. [23]) and eight clusters were found in the genome of *W*. *ichthyophaga* by the antiSMASH software (**Table 2**, **S2 Table**). Such low numbers are in concordance with observations from another xerophilic fungus, *Xeromyces bisporus*, which also has few identifiable secondary metabolite clusters in the genome [16].

**Table 2:** Secondary metabolite biosynthetic gene clusters in *W*. *mellicola* and *W*. *ichthyophaga* identified by the antiSMASH.

Secondary metabolites biosynthetic cluster type	Number per species	
	*W*. *ichthyophaga* (EXF-994) (Zajc et al. 2013b)	*W*. *mellicola* (CBS 633.66) (Padamsee et al. 2012)
Terpene	3	4
Non-ribosomal peptide synthases	1	1
Other	4	4
Total number of clusters	8	9

Most of the gene clusters identified by antiSMASH were present in both investigated *Wallemia* spp., with a high degree of synteny, namely: one cluster of NRPSs, three for the synthesis of terpenes and three with other functionalities. Two clusters were found only in *W*. *mellicola* (type “terpene” and “other”), while one was identified only in *W*. *ichthyophaga*. The latter contained several genes, among them: (a) a tandem duplication of a 4-coumarate-CoA ligase-like gene, (b) genes encoding a protein similar to a copper-transporting ATPase and a drug resistance transporter, (c) a gene for a linear-gramicidin-synthase-subunit-D-like protein, and (d) a lysine-specific histone demethylase 1-like gene.

A Blast search of *W*. *ichthyophaga* and *W*. *mellicola* predicted four homologues of known PKSs in each species. Orthologues from both species were closely related. Phylogenetic analyses of these and similar proteins from the SwissProt and GenBank non-redundant databases showed that one of the proteins from each species belonged to a cluster with quinone oxidoreductases, potentially involved in the oxidation of walleminol into walleminon, while three clustered with carnitine O-acyltransferases.

PKSs are involved in the synthesis of polyketides, a huge family of secondary metabolites, which share the same precursor, acetyl CoA. Two examples of economically important polyketide-derived secondary metabolites are ochratoxin A in *Aspergillus ochraceus* and *Penicillium verrucosum* [57], and citrinin in *Monascus purpureus* [58]. So far no polyketides of *Wallemia* spp. had their structure elucidated, although the presence of acetyl CoA synthetase genes in both species with the sequenced genomes, *W*. *ichthyophaga* and *W*. *mellicola*, support the possibility that *Wallemia* spp. are able to produce polyketide metabolites. Possible candidates for these would be the here observed red pigments or melanin.

Secondary metabolites synthesized by NRPSs in *Wallemia* spp. include indole alkaloids, such as tryptophol and tryptophol acetate [26, 28], which are derived from the amino acid tryptophan. These aromatic alcohols are important signalling molecules that play a significant role in the control of morphogenesis in the fungal cells [59].

The BLAST search for proteins with similarity to NRPSs identified 10 predicted peptides in *W*. *ichthyophaga* and 7 in *W*. *mellicola* (**Table 3**). Among these were putative aminoadipate semialdehyde dehydrogenases, 4-coumarate-CoA ligasesXP_009267576, siderophore peptide synthases, and three proteins with low similarity to siderophore peptide synthases and proteins similar to TdiA, a single-module NRPS from *Aspergillus nidulans*, which is a member of the terrequinone A biosynthesis gene cluster. The diversity of NRPSs was equal or larger in *W*. *ichthyophaga* compared to *W*. *mellicola*. The largest difference was in the number of putative 4-coumarate-CoA ligases, where *W*. *ichthyophaga* had four closely related copies, while *W*. *mellicola* had only one.

**Table 3:** The diversity of proteins similar to polyketide synthases and non-ribosomal peptide synthetases in the genomes of *W*. *ichthyophaga* and *W*. *mellicola* as determined by the psiblast search with homologues from other fungi and confirmed by the phylogenetic analysis.

Function of most closely related homologues from GenBank NR database	*W*. *ichthyophaga* proteins	*W*. *mellicola* proteins
carnitine O-acyltransferase	XP_009269592, XP_009267950, XP_009265813	XP_006960470, XP_006955789, XP_006957022
aminoadipate semialdehyde dehydrogenase	XP_009270199	XP_006960662
4-coumarate-CoA ligase	XP_009269669, XP_009267575, XP_009267576, XP_009270415	XP_006958156
acetyl CoA synthetase	XP_009270293	XP_006957719
siderophore peptide synthases (NRPSs)	XP_009269655	XP_006955904
unknown NRPSs, sharing some similarity to siderophore peptide synthases	XP_009270084, XP_009269961, XP_009267579	XP_006960464, XP_006960519
TdiA (*Aspergillus nidulans*)	XP_009269410	XP_006957811
quinone oxidoreductase	XP_009268752	XP_006960148

Wallemia A and Wallemia C may be biosynthesized via proteins encoded by both NRPS and terpene-unit gene clusters. Furthermore, the production of terpene derived secondary metabolites might also involve NRPSs. Two terpene derived sterols UCA 1064-A and UCA 1064-B reported from *Wallemia* spp. contain an unusual N atom, probably derived from an amino acid. Other observed terpenes include walleminon and walleminol, which possibly act as sporulation hormones, analogously to endogenous diterpenes conidiogenone and conidiogenol found in penicillia [60, 61].

### Predictive clustering trees revealed key features influencing the production of secondary metabolites in *Wallemia*

To further investigate the secondary metabolite production by seven *Wallemia* spp. we grew 30 strains, isolated from indoor air and diverse low a_w_ environments, on YES, CYAS and WATM. As reported previously, the growth parameters of *W*. *ichthyophaga* differed from other species. Although *W*. *ichthyophaga* grew optimally at 15% NaCl, growth was observed also at 20% and 25% NaCl, and even at 11% and 17% MgCl_2_ (**S1 Table)**. After incubation and extraction, culture extracts were screened for the presence of secondary metabolites. Approximately one hundred compounds were detected by HPLC-DAD and characterized by their provisional names, retention times, and characteristic UV/VIS spectra (**S3 Table**). All peaks were quantified by height and the data were collected in an Excel file (**S4 Table**) for machine learning methods.

**Fig 1:**
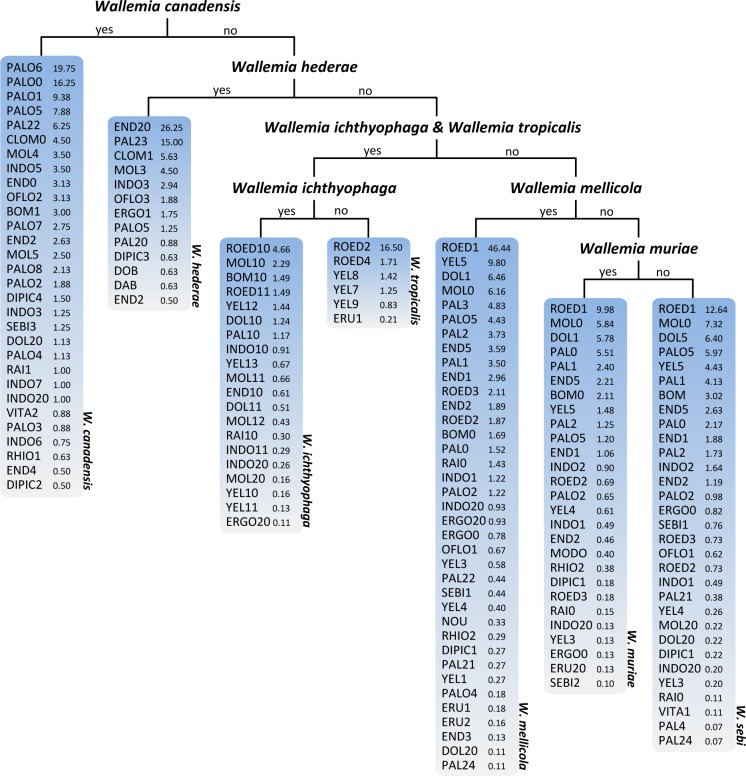
Visualization of the predictive clustering tree (PCT) of secondary metabolites in *Wallemia* constructed by using only the species itself as a descriptive variable. As target variables the quantity of the 96 secondary metabolites were used. Each of the seven leaves make a prediction of secondary metabolites produced by individual species. In each leaf, the predicted quantities of the secondary metabolites are listed.

**Fig 2:**
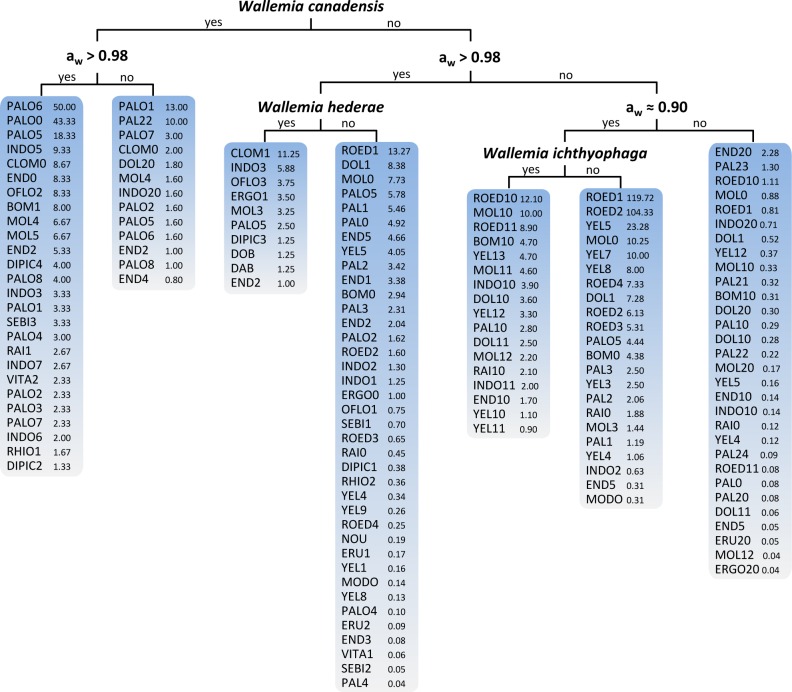
Visualization of the predictive clustering tree (PCT) of secondary metabolites in *Wallemia* constructed by using the species itself and the water activity as a descriptive variable. As target variables, the quantities of the 96 secondary metabolites were used. In each leaf, the predicted quantities of the secondary metabolites are listed.

**Fig 3:**
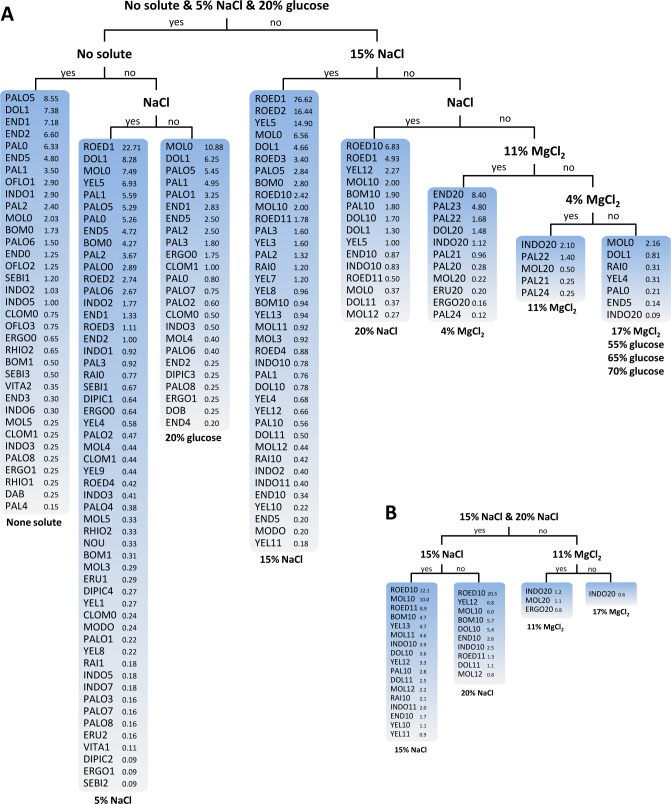
Visualization of the predictive clustering tree of secondary metabolites in *Wallemia* constructed by using the solute type and the solute concentrations as descriptive variables. As target variables the quantities of the secondary metabolites were used: (A) for all species and (B) for *W*. *ichthyophaga*, separately. The internal nodes contain tests on individual conditions (e.g., 15% NaCl) and leaves correspond to a specific combination of conditions. In each leaf, the predicted quantities of the secondary metabolites are listed (sorted descending).

The decision trees (more specifically PCTs) obtained by machine learning analysis in various scenarios revealed key features influencing the production of secondary metabolites in *Wallemia* (**Figs 1–3** and **S1 File**). The PCTs are easily interpretable predictive models. Detailed information about predictive clustering trees for multi-target regression has been published before [51–54].

When the species itself (scenario A, see the Materials and Methods section) was used as the only descriptive variable, each of seven leaves illustrates the profile of secondary metabolites for each of the species separately. Reaching the top position of the PCT, *W*. *canadensis* exhibited the most unique secondary metabolism, followed by *W*. *hederae*, *W*. *ichthyophaga* and *W*. *tropicalis*. In comparison to other species, *W*. *tropicalis* synthetized a relatively small number of secondary metabolites. Finally, the remaining three species (*W*. *sebi*, *W*. *mellicola* and *W*. *muriae*) had relatively similar secondary metabolite profiles. These results indicate PCTs are a useful new tool for the analysis of secondary metabolite chemotaxonomy. Chemical diversity as a taxonomic tool [62] was previously applied in the taxonomic revision of the *W*. *sebi* species complex [23]. The differences in metabolite production reflect the phylogenetic distance of *W*. *canadensis* compared to the close relatedness of the species *W*. *sebi*, *W*. *mellicola* and *W*. *tropicalis*.

In addition to the species itself, a_w_ of the media, where cultures were grown, turned out to be the key feature influencing the production of secondary metabolites in *Wallemia* spp. (**Fig 2**, scenario B). Comparable to the tree from scenario A, the most limiting information was the species itself, with *W*. *canadensis* at the top of the decision tree. The next limiting feature was high a_w_ (> 0.98) that is usually favourable for the growth and physiological activity of microorganisms, including biosynthesis of secondary metabolites [63]. *Wallemia canadensis* and *W*. *hederae* grown on media with a_w_ higher than 0.98 had an overall higher production of secondary metabolites, than on media with lower a_w_. Only *W*. *ichthyophaga* had the most prominent production of secondary metabolites when grown on media with lower a_w_ (≈ 0.90). In all other species besides *W*. *ichthyophaga* even a_w_ < 0.98 decreased the overall production of secondary metabolites.

The PCT presented in **Fig 3A** was constructed using the solute types (glucose, NaCl, MgCl_2_) and the solute concentrations as descriptive variables, independent of the species itself. NaCl in various concentrations (0%, 5%, 15%, 20% and 25%) had the greatest influence on the production of secondary metabolites in *Wallemia* spp. This is a strong indication that the growth and secondary metabolism in *Wallemia* spp. are not influenced only by the reduction of a_w_, but also by the type of the solute. Although all *Wallemia* species were previously characterized as halophilic or, at least, halotolerant [19, 23, 24, 47, 48], little was known about the production of metabolites when exposed to salt stress. Only Peng et al. [17] reported on a series of mainly aromatic compounds, isolated from a marine strain of *W*. *sebi* grown at 10% NaCl (w/v). Furthermore, the HPLC profile of the extract from culture grown at 10% NaCl differed from the extracts grown on media without and with 3% NaCl [17].

Results presented in the decision tree in **Fig 3A** indicate that when no NaCl was added to the growth medium, the most abundantly produced secondary metabolites by *Wallemia* spp. (with the exception of *W*. *ichthyophaga*, which does not grow without the addition of salt) were PALO5, DOL1, END1, END2 and PAL0. With the addition of 5% NaCl other secondary metabolites prevailed: ROED1, DOL1, MOL0 and YEL5 (with ROED1 dominating over the others). The addition of 5% NaCl to the growth medium triggered a production of a much higher number of secondary metabolites than the addition of 20% glucose or no solute, while the addition of 15% NaCl resulted in a very large quantity of ROED1 and large quantities of ROED2 and YEL5. Further increase of NaCl concentration to 20% resulted in increased quantities of ROED10.

The PCTs constructed for each species separately by using the solute type and solute concentration as descriptive variables are depicted in **Fig 3B** and **S1 File**. *Wallemia ichthyophaga* is an obligate halophile, which requires at least 9% NaCl for *in-vitro* growth and it thrives even at saturated NaCl concentrations [37, 47]. Available strains of this species so far were isolated from salted meat, hypersaline water, sea salt crystals, bitterns and, surprisingly, from the air in horse stables and hay barns in Denmark [19, 24]. The results presented on **Fig 3B**, which gives the PCT constructed for *W*. *ichthyophaga* are in accordance with its halophilic nature. The largest quantities of secondary metabolites were produced at 15% NaCl in the growth media. The ROED10 was the most prominent secondary metabolite and its quantity at 20% NaCl increased even further.

**Fig 4:**
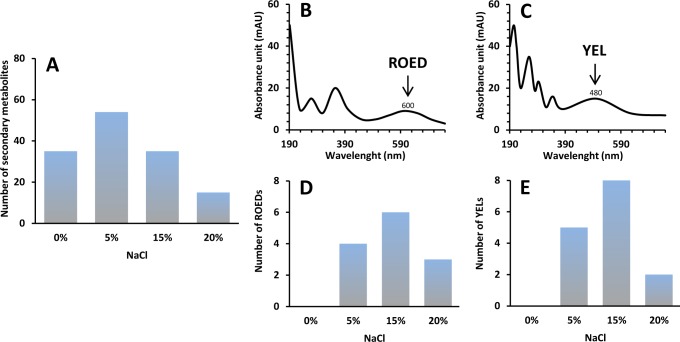
Influence of NaCl on the number of secondary metabolites produced by *Wallemia*. Number of secondary metabolites in total (**A**), ROEDs (**D**) and YELs (**E**) on media with different NaCl concentrations (0%, 5%, 10%, 15% and 20%). **B-C**. Spectrum of ROED (**B**) and YEL (**C**) with characteristic peaks at 600 nm and 480 nm, respectively.

In the performed experiments the addition of NaCl to the growth media stimulated the total production of secondary metabolites in all *Wallemia* spp. (**Fig 4A**), and in particular the production of metabolites from the ROED and YEL family. ROEDs and YELs are provisional names for red and yellow pigments with the characteristic UV/VIS peaks at 600 nm and 480 nm, respectively (**Fig 4B** and **4C**). ROEDs and YELs were not produced in media with glucose or media without NaCl, while the addition of 5% and 15% NaCl clearly increased their total number. These compounds were detected even at 20% NaCl (**Fig 4D** and **4E**), indicating that these pigments might play a role in the protection of *Wallemia* cells at high salt stress conditions. Pigments are known to play an important role in the survival of microbes at hypersaline conditions, whether in algae (*Dunaliella salina*) [64], black yeasts (*Hortaea werneckii*) [65, 66], Archaea (species of genera *Haloarcula*, *Haloferax*, *Halorubrum* and *Halobacterium*) and Bacteria (*Salinibacter ruber*) [67].

The UV/VIS spectra of ROEDs and YELs indicate that the compounds are polyketide-derived anthraquinone pigments possessing a conjugated chromophore. In addition, melanin is also polyketide derived and it also protects mycelium and conidia from UV and other irradiations [68]. As we noted previously, the putative gene coding for a PKS was found in the genome of *W*. *ichthyophaga* and can be assumed to be the key gene in the production of these pigments.

In **S1 File** the PCT for the halotolerant species *W*. *hederae*, the phylogenetic sister species of the halophilic *W*. *ichthyophaga* is presented. Even though it grows optimally, like *W*. *ichthyophaga*, at 15% NaCl [24], surprisingly only one secondary metabolite (MOL3) was detected at this salinity. Addition of 5% NaCl yielded 5 metabolites, with CLOM1 and MOL3 in the largest quantities. The most diverse range of metabolites (8) was synthesized when no solute or 20% glucose were added to the growth media, indicating that the species should be regarded more as an osmophile than halophile. In comparison to *W*. *ichthyophaga* that was isolated primarily from hypersaline habitats, *W*. *hederae* was isolated so far primarily from plant associated substrates [24].

One of the most important habitats for *Wallemia* spp. are bitterns and hypersaline water of solar salterns. Here they are not only subjected to low water activity, but also to high concentrations of chaotropic (MgCl_2_) and kosmotropic (NaCl, MgSO_4_) stressors [69–71]. Recently it was demonstrated that unlike any prokaryote described so far, *W*. *sebi*, *W*. *mellicola*, *W*. *canadensis*, *W*. *tropicalis*, *W*. *hederae* and *W*. *muriae* can grow at MgCl_2_ concentrations of up to 11%-17%, while *W*. *ichthyophaga* can grow even up to 20% MgCl_2_ [23, 24, 72]. Addition of the chaotropic MgCl_2_ as solute to the growth media resulted either in very small production of secondary metabolites or even their complete absence. Eleven percent of MgCl_2_ yielded larger quantities of END20 and PAL23. At 17% of MgCl_2_ only *W*. *ichthyophaga* synthesized a small quantity of INDO20, which could be tryptophol acetate, as INDO20 has a tryptophan chromophore. To the best of our knowledge there are no published studies on the production of secondary metabolites at chaotropic conditions (MgCl_2_) either by fungi or by prokaryotes.

### Analysis of biologically significant metabolites with mass spectroscopy

So far, a total of approximately 25 metabolites have been isolated and identified in *Wallemia*. We selected five biologically active metabolites for mass spectroscopy analysis: wallimidione (monoisotopic mass 244.12 Da), walleminone (252.17 Da), walleminol (236.18 Da), UCA 1064-A (411.35 Da) and UCA 1064-B (413.37 Da). The peaks of wallimidione, walleminone, walleminol, UCA 1064-A and UCA 1064-B occurred at around 4.48, 8.45, 8.78, 9.93 and 1.46 min, respectively (**Table 4**). Each metabolite is discussed below.

**Table 4:** Species specific production of biological significant secondary metabolites wallimidione, walleminone, walleminol, UCA 1064-A and UCA 1064-B.

Metabolite	Wallimidione	Walleminone	Walleminol isomer	UCA 1064-A	UCA 1064-B
Measured retention time (RT, MEAN ± standard deviation)	5.48 ± 0.01	8.54 ± 0.01	8.78 ± 0.01	9.92 ± 0.02	10.4 ± 0.02
*Wallemia sebi*	+	+	+	-	+
*Wallemia mellicola*	+	+	+	+	+
*Wallemia canadensis*	+	+	+	+	+
*Wallemia tropicalis*	+	-	-	+	+
*Wallemia muriae*	+	+	+	+	+
*Wallemia ichthyophaga*	+	-	-	+	+
*Wallemia hederae*	+	-	-	-	+

#### Wallimidione

**Fig 5:**
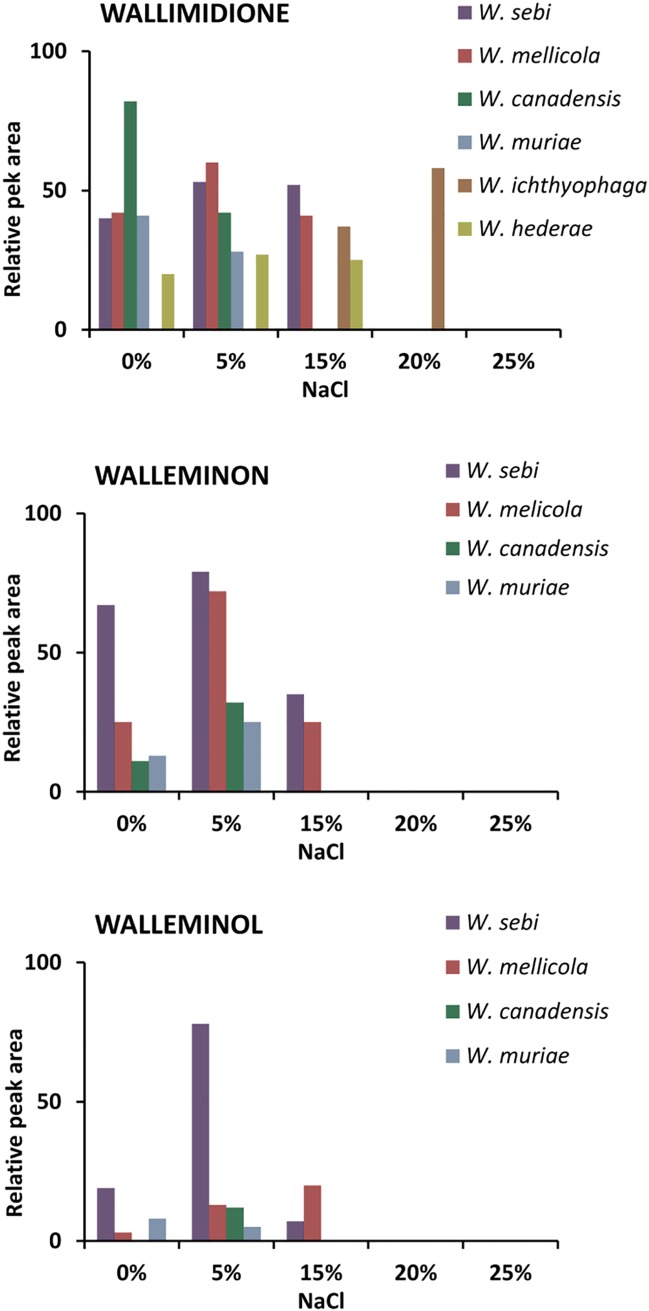
Production of wallimidione, walleminone and walleminol across the NaCl gradient.

Based on the Toxtree analysis [73], wallimidione is the most toxic of the metabolites reported to date from *Wallemia*. Isolation and characterization of this metabolite was carried out with an indoor isolated culture of *W*. *sebi* group 3 [26] that was later described as the new species *W*. *canadensis* [23]. According to our mass spectroscopy analyses, all species from the genus *Wallemia* produced wallimidione (**Table 4**), meaning it has no chemotaxonomic differentiation power within the genus *Wallemia* [62]. Its production is, however, strongly affected by NaCl (**Fig 5**). An increase in NaCl concentration up to 15% in the growth media increased wallimidione production by *W*. *sebi*, *W*. *mellicola*, and *W*. *hederae*, while the production of this metabolite by the obligately halophilic *W*. *ichthyophaga* was largest at optimal salinities from 15% to 20% NaCl.

#### Walleminol and walleminone

Historically, *W*. *sebi* was first shown to produce toxic metabolites during a toxilogical screening of fungi isolated from mouldy food [29]. Walleminol and walleminone are sesquiterpene caryophyllenes, a class of terpenes that consist of three isoprene units and have a molecular formula C_15_H_24_ [28]. Genomic data of both *W*. *mellicola* and *W*. *ichthyophaga* revealed four and three terpene biosynthetic clusters, respectively. However, walleminol and walleminone were detected only in *W*. *sebi*, *W*. *mellicola*, *W*. *canadensis* and *W*. *muriae*, but not in *W*. *ichthyophaga*. A possible reason for this could be the unique cluster for terpene biosynthesis, which is present in the genome of *Wallemia mellicola*, but absent from *W*. *ichthyophaga*. Of all *Wallemia* spp., *W*. *sebi* was the best producer of both walleminol and walleminone (confirmed by mass spectroscopy), followed by *W*. *mellicola* (**Table 4** and **S5 Table**).

Again, just as in the case of wallimidione, an increase in NaCl concentration to 5% in the growth media clearly increased the production of both walleminol and walleminone by all four producing species (**Fig 5**).

#### UCA1064-A and UCA1064-B

The metabolites with known antitumor activities, UCA1064-A (previously designated as A25822B [30]) and UCA1064-B are compounds with unique azahomosterol skeleton, differing in the reduction of C-24 in exomethylene [31]. Candidate genes (terpene clusters) for their biosynthesis were found in sequenced genomes of both *W*. *mellicola* and *W*. *ichthyophaga*. In this study, UCA1064-B was detected in all known species by mass spectrometry, while UCA1064-A was not produced by *W*. *sebi* and *W*. *hederae* (**Table 4**). *W*. *ichthyophaga* strain EXF-6070 isolated from bitterns [24] produced both metabolites even at 20% NaCl.

## Conclusions

Secondary metabolites are consistently produced by *Wallemia* spp. and their production is–contrary to common presumptions–increased as a response to NaCl concentration. Accordingly we suggest that these metabolites play a role in the adaptation to hypersaline conditions, particularly in *W*. *ichthyophaga*. Accumulation of small protective metabolites such as melanin, mycosporines, mycosporine-like amino acids or carotenoids (as well as other substances) is known to be important in the survival of extreme conditions, as reviewed by Gostinčar et al. [14]. In addition to protecting from UV and even ionizing radiation, melanin was for example suggested to impermeabilize the cell wall and reduce the leakage of compatible solutes from the cell [74], mycosporines possibly function as compatible solutes [75], and all of these compounds (as well as several others) also scavenge reactive oxidative species [14] and thus alleviate the secondary oxidative stress triggered by other stress factors (salinity being one of them). The possible roles of the *Wallemia* spp. secondary metabolites in inter-species interaction should also be considered, especially for compounds that are active against other organisms. It is true that the extreme conditions of the *Wallemia* spp. habitats support the growth of few other species. However, the scarce diversity (and the fact that other species are often present in large numbers due to decreased competition), make any interaction with them all the more important for the survival of *Wallemia* spp. From a more applicative perspective, the consequences of the mycotoxigenic potential of *Wallemia* spp. for food, feed and air quality should be considered. We showed that these species can not only produce toxic metabolites walleminol, walleminone and wallimidione, but also that this production is not repressed at high-salt conditions, like it is the case with most other mycotoxigenic fungi–in many cases it is even induced. This exception to the rule makes *Wallemia* spp. a potentially serious and currently largely neglected health risk associated with salt-preserved food and other scenarios involving high concentrations of salt. This warrants further research, which should include absolute quantification of walleminol, walleminone and wallimidione, tryptophan derived secondary metabolites and unknown compounds detected in *Wallemia* spp. as well as further toxicity tests to provide conclusive evidence on their impact on vertebrates in concentration expected in real-life settings.

## Supporting Information

S1 File PCTs constructed for each species separately by using the solute type and solute concentration as descriptive variables. (PDF)

S1 Table List of analyzed strains, their sources, and size of their colonies grown on YES, CYAS, and WATM with NaCl, glucose and MgCl_2_ added in different concentrations at 25°C in the dark after 10 days. (XLSX)

S2 Table Secondary metabolite biosynthetic homologous gene clusters in *W*. *mellicola* and *W*. *ichthyophaga*. (XLSX)

S3 Table Secondary metabolites detected by HPLC-DAD analysis. (XLSX)

S4 Table Dataset from HPLC-DAD analysis for machine leraning analysis. (XLS)

S5 Table Detection of wallimidione, walleminone, walleminol, UCA 1064-A and UCA 1064-B by mass spectroscopy. (XLSX)
