# Supplementary figures and images for: Production of Secondary Metabolites in Extreme Environments: Food- and Airborne Wallemia spp. Produce Toxic Metabolites at Hypersaline Conditions
Source: PLoS One. 2016 Dec 30;11(12):e0169116. doi: 10.1371/journal.pone.0169116 (PMC5201246; doi:10.1371/journal.pone.0169116)

W. hederæ

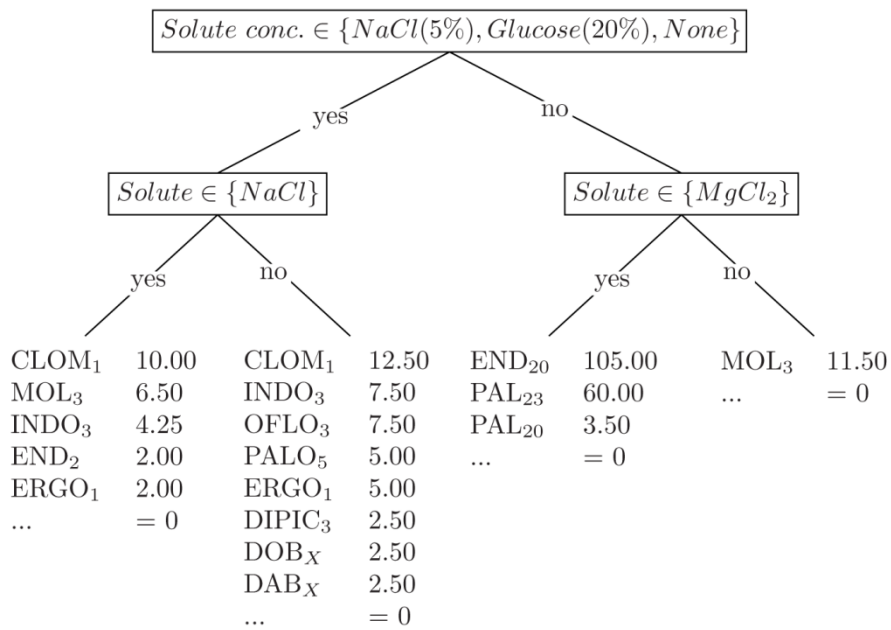

W. sebi

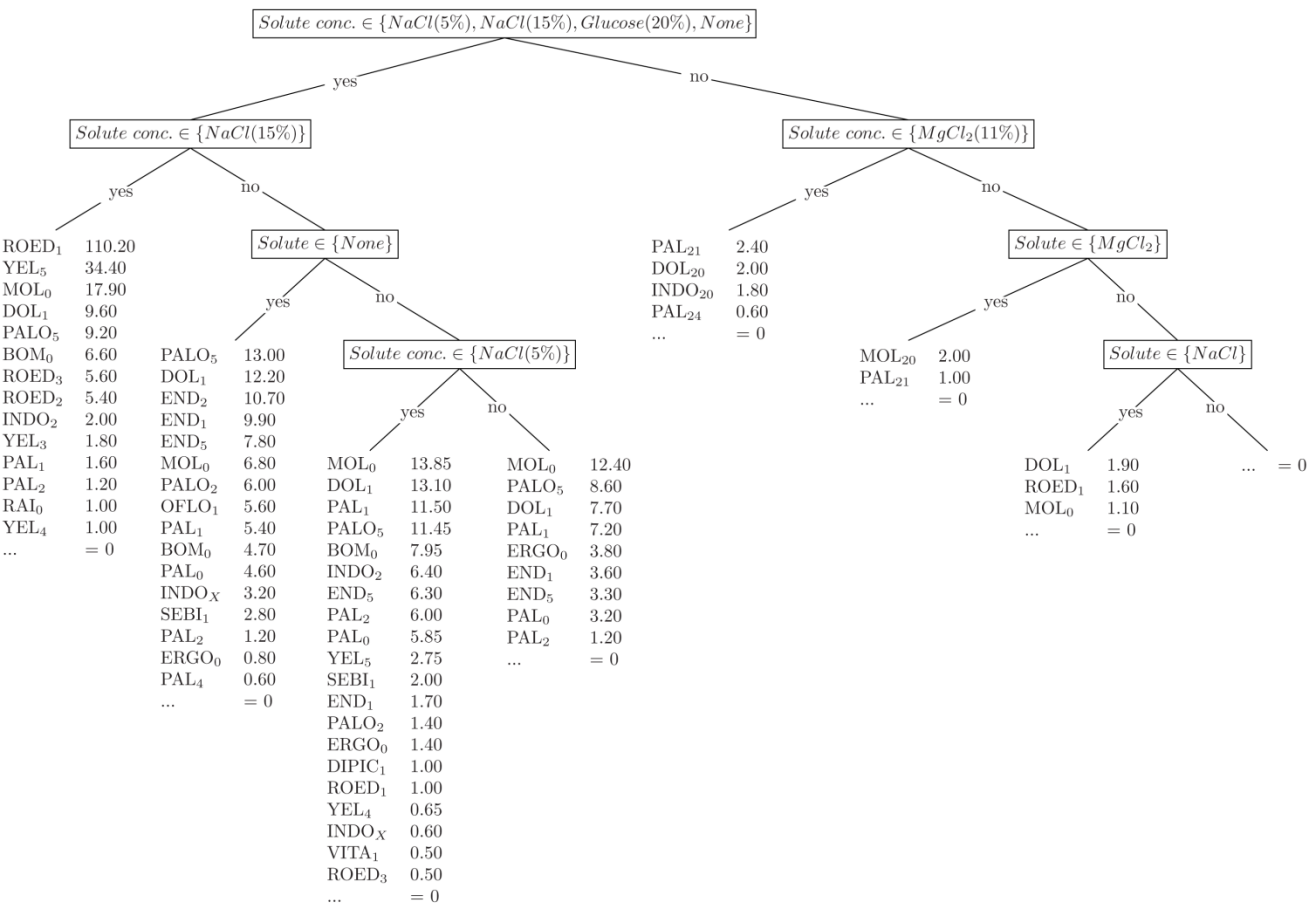

W. mellicola

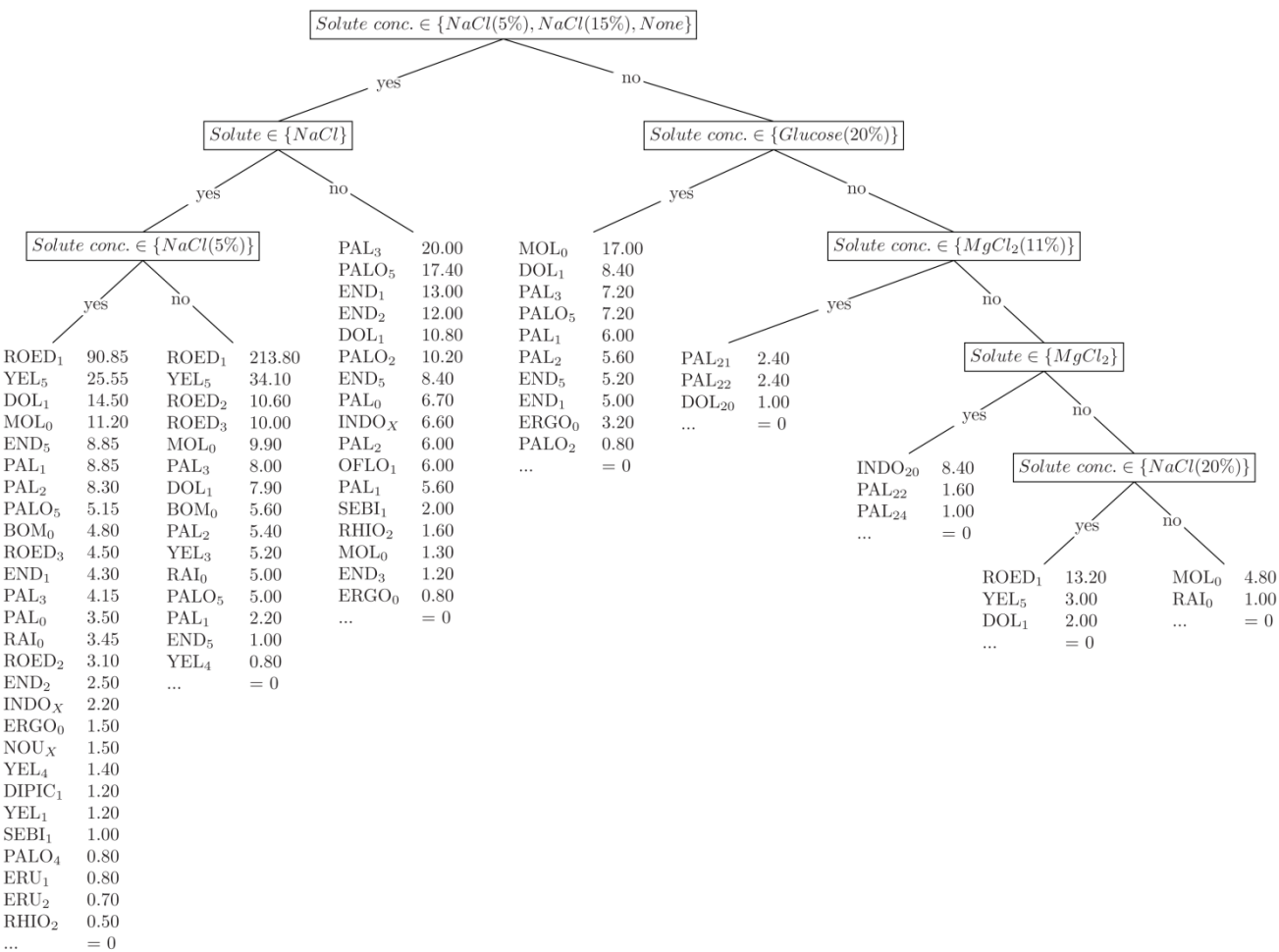

W. canadensis

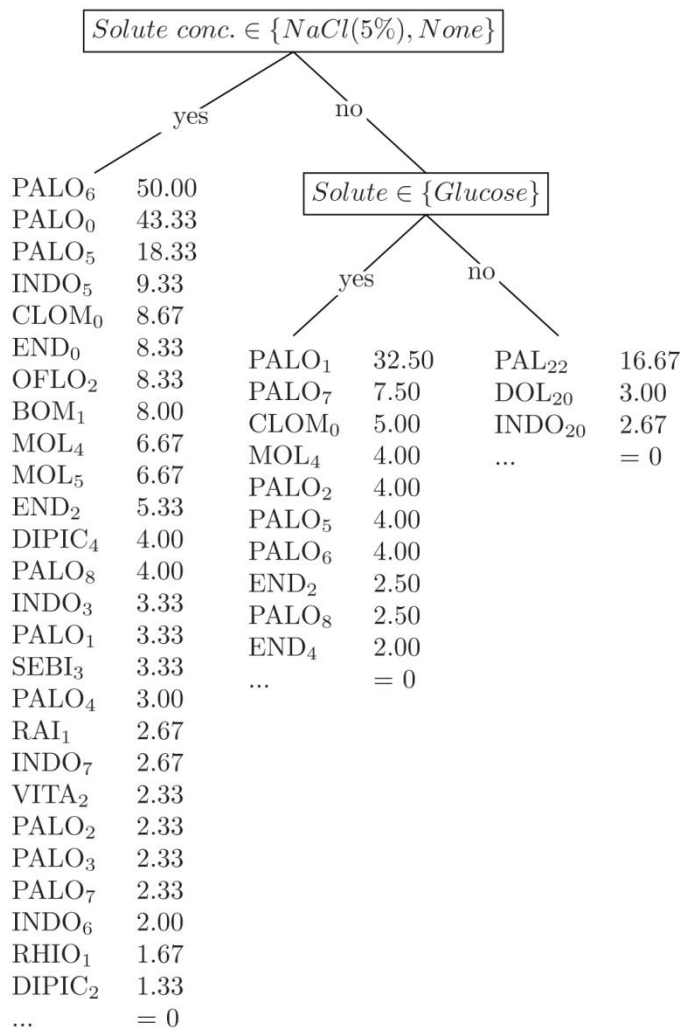

W. muriae

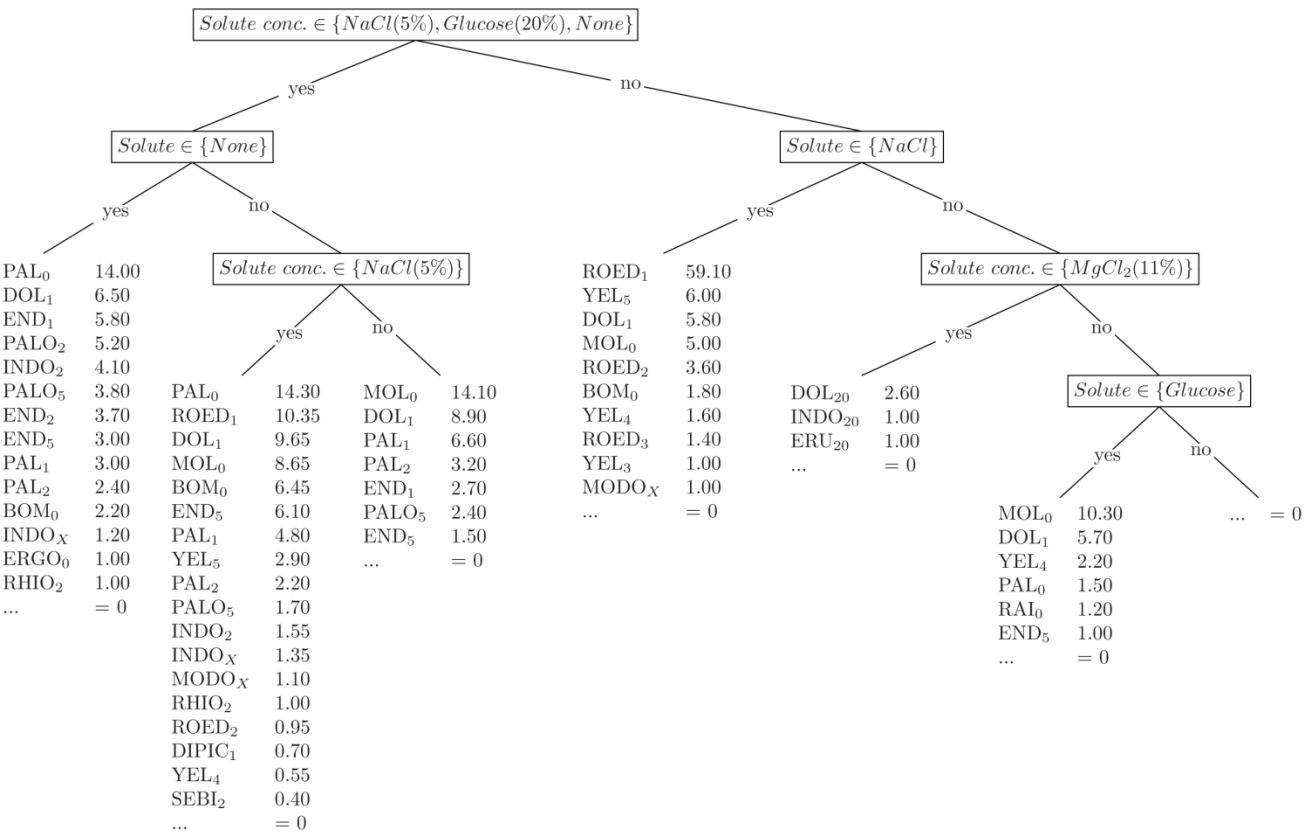

*W. tropicalis*

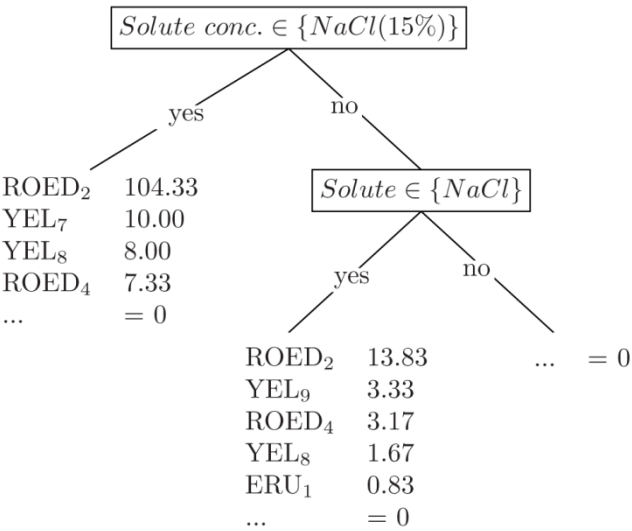

Supplement: S1 File — (PDF) [file pone.0169116.s001.pdf]
